# Association Between Intergenerational Support, Social Integration, and Subjective Well-Being Among Migrant Elderly Following Children in Jinan, China

**DOI:** 10.3389/fpubh.2022.870428

**Published:** 2022-06-10

**Authors:** Qingchen Jia, Shixue Li, Fanlei Kong

**Affiliations:** ^1^Centre for Health Management and Policy Research, School of Public Health, Cheeloo College of Medicine, Shandong University, Jinan, China; ^2^NHC Key Lab of Health Economics and Policy Research (Shandong University), Jinan, China

**Keywords:** migrant elderly following children, intergenerational support, social integration, subjective well-being, hospitalization

## Abstract

**Background:**

In China, as domestic urbanization progresses and immigration expands, an increasing number of older people are choosing to follow their migrant children to new cities. Such people are referred to as “migrant elderly following children.” However, few studies have explored the subjective well-being of these older adults. The present study aims to investigate the factors that influence the subjective well-being of this population of older adults.

**Methods:**

This cross-sectional study was conducted among 656 older migrants who had followed their children to Jinan, Shandong Province, China. Multistage cluster random sampling was used. Binary logistic regression analyses were performed to explore, the relationships between intergenerational support, social integration, and subjective well-being.

**Results:**

Overall, 96.3% of the older migrants showed good subjective well-being. Analysis of intergenerational support showed that those who had a female child (odds ratio [OR] = 0.401, 95% confidence interval [95%CI]: 0.180, 0.893) and those whose children had terrible conjugal relationships (OR = 0.223, 95% CI: 0.099, 0.504) were less likely to have better subjective well-being. Analysis of social integration showed that migrants who liked their current city (OR = 5.358, 95%CI: 1. 631, 17.599) and those who had a basic understanding of the local dialect (OR = 2.869, 95%CI: 1.203, 6.843) were more likely to have good subjective well-being. Migrants who had used in-patient service in the past year (OR = 0.216, 95%CI: 0.094, 0.497) were more likely to have poor subjective well-being.

**Conclusion:**

Intergenerational support and social integration are positively associated with the subjective well-being of migrant elderly following children in China. Efforts should be made, including the creation of specialized policies, to improve the family atmosphere of such migrants and their integration into their new cities, as this would contribute to improving their subjective well-being.

## Introduction

Over the past 10 years, China's economy and health-care system have developed rapidly, while population aging, urbanization, and population mobility have also steadily increased (1). This huge economic improvement has driven large-scale migration from economically undeveloped areas (2), with the number of internal migrants increasing from 21.35 million in 1990 to 247 million in 2015 (3). The flow of Chinese migrants has now changed from a period of rapid growth to a period of adjustment, and family migration now (and for the foreseeable future) represents the main trend in population mobility (4, 5). Older adults who have migrated with their children are known as “migrant elderly following children” (MEFCs) (3). When MEFCs migrated to a new place, the fragility of them would increase (6). Specifically, the mental state and emotional needs of the MEFCs would be influenced due to the change of living conditions and social integration (4, 7). Unfortunately, MEFCs had obstacles in accessing local health care services because they were unfamiliar with the local medical institutions (8). In addition, considering the high costs and inconvenience in obtaining insurance reimbursement, MEFCs might be limited to access the health care services (9). However, there were few studies pay attention to MEFCs and their mental health and well-being.

Subjective well-being (SWB) represents a person's subjective assessment of their quality of life, happiness, and satisfaction, as well as the quality of their experiences with regard to other areas of life (10–12). SWB can be divided into several dimensions: life satisfaction (global judgments of one's life), satisfaction with important domains (e.g., work satisfaction), positive effect (regularity of experiencing pleasant emotions and moods), and negative effect (regularity of experiencing unpleasant emotions and moods) (13–16). A study found that migrants who live in Southern Europe, Eastern Europe, and non-European countries have significantly lower levels of SWB than the native populations (17). Further, a seminal study in this area similarly found significant differences between migrants and natives in Germany, identifying migrants from Southern Europe, Turkey, and former Yugoslavian countries as the groups with the lowest SWB levels (18). However, most previous studies on the SWB of migrants in China have focused only on migrant workers, ignoring the social integration and emotional state of the more vulnerable older migrants (19, 20). In particular, few studies have focused on the SWB of MEFCs.

Intergenerational support in this study refers to the support adult children provide to their older parents. The present study measured intergenerational support mainly by considering children's provision of daily care and emotional support to their older parents (21–23). Previous studies have shown that financial support from children was an important economic resource, which can positively affect the parents' SWB. Moreover, these two main dimension of intergenerational support conforms to the traditional Chinese norm of filial piety (24, 25).

Li et al. showed that the quality of the relationship between older adults and their adult children, older adults' satisfaction with their adult children, and the happiness of one's adult children affect the SWB of older adults (26). Wang also found that receiving care from one's offspring and the quality of one's intergenerational relationships influence the SWB of older adults (25). Chen assessed the impact of monetary support from one's children, demonstrating that these benefits have positive effects on the economic status and SWB of older adults (27). A study by Giles concerning intergenerational support among migrants found that, in China, rural-based older adults with migrant children receive less support than those who did not have migrated children in the form of both income and in-kind instrumental care (24). Finally, He et al. highlighted that intergenerational support for older adults is decreasing both in structure and scale, with family monetary support also weakening (28). Previous studies have mainly focused on the role of economic support in SWB, and have not found a relationship between emotional support and SWB.

Social integration is a multidimensional concept, and represents the degree to which individuals participate in a wide range of social roles and relationships (29), as well as their acceptance of cultures and identities (29–31). One study reported that migrants who have left their habitual residences show higher SWB when they feel socially integrated into their current cities (32). Another article highlighted that acculturation and identification are key dimensions of social integration, and play a critical role in the development of migrants' SWB (33). Existing research on migrants has focused mainly on economic aspects, and there has been relatively little empirical research on migrants' cultural, behavioral, and psychological identity. There is also a lack of empirical research in areas such as language adaptation, especially among MEFCs (34). Because many MEFCs must adapt to new environments with limited social support. Moreover, MEFCs would face more difficulties than younger migrants as their health and financial resources also decline.

Theoretical discussion on social integration among migrants can be divided into the three major schools of “assimilation theory”, “multiculturalism” and “segmented assimilation”. Assimilation theory was one of the widely used theories in migration-related studies, which could explain acculturation, identification, economic integration, and behavioral adaption. In this study, we mainly discussed the acculturation and identification of MEFCs as two dimensions to assess social integration (35). We adopted the theoretical framework of social integration based on previous findings (31), and used the same two dimensions to explore the association between acculturation, identification, and SWB.

The present study mainly aimed to investigate the association between social integration, intergenerational support, and SWB among MEFCs. And our findings may improve the understanding of MEFCs. It is contributable to identify the methods of facilitating positive and progressive development among MEFCs.

## Materials and Methods

### Study Setting

The data for this research were collected in Jinan City, Shandong Province, China, in August 2020. Shandong Province lies in the east of China. Jinan City is the capital city of Shandong Province, and its gross domestic product in 2020 was 1.01 trillion RMB. As of July 1, 2020, Jinan has 10 districts and two counties (132 sub-districts and 29 towns) under its jurisdiction (36). By the end of 2019, the resident population was 8.91 million, an increase of 0.78% over the previous year, while the registered population was 7.98 million, an increase of 1.46% from the previous year (37). There were 2.9 million migrants in Jinan City in 2019, of whom those who were older than 60 years and who had followed their children to Jinan City were the target population for this study.

### Data Collection

Multi-stage cluster random sampling was used to select participants for this study. In the first stage, two districts were chosen from Jinan's 10 districts to represent the primary sampling units (PSUs). In this selection, the economic development and geographic location of each district were considered. In the second stage, two sub-districts were selected from each PSU to serve as secondary sampling units (SSUs), meaning one sub-district was chosen from each of the previously selected districts. In the third stage, two communities were selected from each SSU, meaning one community was chosen from each of the previously selected sub-districts. All older migrant adults in these two communities who were aged over 60 years and who had followed their children to Jinan City constituted the final target sample for this study. Thirty-two university students were recruited to act as investigators and, prior to performing data collection, received training that included background information concerning the study, information on the contents of the questionnaire, and instruction on techniques for performing social surveys. Eleven of the investigators were from Shandong University, 13 were from Jinan University, two were from Dongying Vocational Institute, and seven were from Weifang Medical University. To collect the data, the investigators conducted 20-min face-to-face interviews with subjects. A total of 670 MEFCs were initially chosen and interviewed. However, 14 of these were excluded from the sample due to obvious logical errors in questionnaire responses or incomplete responses. Thus, data for a total of 656 older individuals were included in the database.

### Variables

#### Sociodemographic Characteristics

The survey collected sociodemographic information from the participants, including age, gender, marital status, hukou (38), education level (39), duration in Jinan, migration scale (40), migration space range (33), income (41, 42), etc. Hukou is a household registration system used in China, with individuals being assigned rural or urban hukou based on their place of birth and lineage (Rural = 0, Urban = 1). Migration scale (Alone = 0, With one family member = 1, With two family members or more = 2) (43). Migration space range referred to the distance from MEFC's hometown to the current city (Trans-counties = 0, Trans-cities = 1,Trans-provinces = 2, Trans-countries = 3) (41).

#### Subjective Well-Being

The dependent variable in this study, SWB, was measured by asking the respondents: “What is your current level of happiness?” Participants could provide four possible responses: “very high,” “high,” “low,” and “very low,” with “low” and “very low” denoting poor SWB and “very high” and “high” denoting good SWB (44, 45). In an examination of this self-report inquiry method, Wilson compared respondents' answers with expert assessments of the respondents and found that this method produces high consistency in most cases (46). Therefore, the self-report method used in this study was deemed reliable and valid for measuring SWB.

#### Intergenerational Support

This research considered only two types of intergenerational support: daily care and emotional support. To measure the daily care dimension, we asked respondents to report the gender of their children (Male = 0, Female = 1) and whether their children worked overtime in the home (Often = 0, No or less = 1). The gender of one's child has been shown to have a significant impact on daily care for older people (47–49), while Liu showed that children who work long hours have less time to devote to daily care, thereby negatively impacting care quality (50). To measure the emotional support dimension, we asked respondents to report on the quality of their children's conjugal relationships (Good = 0, Terrible = 1). Previous research has shown that the better a child's conjugal relationship, the more support he/she gives his/her parents (26, 51).

#### Social Integration

In this study, social integration was mainly determined by focusing on respondents' identification and acculturation (52). Identification was defined as “the individual recognizes his or her identity as belonging, affirms the values and behaviors of that particular group and leads to activities that are congruent with the identity” (53), and was mainly measured through the question “Do you favor your current city?” (54). The answer included “No” and “Yes”. Acculturation was measured by asking “Do you understand the local dialect in current city” (Fully understand = 0, Basic understand = 1, Do not understand = 2) (55, 56). The domiciliary transfer was the change of migrants' hukou, usually referred to as rural hukou became urban hukou when migrants lived in the current city (57). Community participation was conceptualized as utilizing community services, participating in community and recreational programming, and other forms of interactions with different community settings (58).

#### Utilization of Medical and Health Services

In this study, utilization of medical and health services was mainly measured by asking participants: “Have you used in-patient service in the past year?” (No = 0, Yes = 1). Medical return was referred to MEFCs return to their hometown to have in-patient service (59).

### Statistical Analysis

All statistical analyses were performed using IBM SPSS 24.0 (Armonk, NY: IBM Corp.), and a bilateral *P*-value of <0.05 was considered to indicate statistical significance. The chi-square test was used to calculate the differences in SWB among the subgroups of each categorical variable. After univariate analyses, statistically significant variables were included in the logistic regression analyses. Three binary logistic regression models with an enter method were employed to explore the associations between the independent variables (intergenerational support and social integration) and migrants' SWB. Specifically, MEFCs' intergenerational support was entered into Model 1. Then, the indicators of social integration were included with MEFCs' intergenerational support to create Model 2. Finally, Model 3 comprised the variables from Model 2 along with respondents' utilization of medical and health services. Odds ratios (ORs) and 95% confidence intervals (95%CIs) were calculated to assess changes.

### Ethical Considerations

Informed consent for the data collection and the use of the data was obtained from all subjects. Ethical clearance was approved by the Shandong University ethics committee (No. 20180225).

## Results

### Participants' Demographic Characteristics

Table 1 shows the demographic characteristics of the 656 participating MEFCs. Most were female (63.7%), were aged 63–65 years (30%), were married (88.9%), had rural hukou (87.5%), had migrated from other cities in Shandong (67.2%), were illiterate (29.9%), had migrated with one family member (60.1%), and had an income of 0–100 yuan per month (33.7%, Table 1).

**Table 1:** Social demographic characteristics among migrant elderly following children.

**Variables**	***N***	**%**	**Migrants' SWB**	
			**Yes**	**No**
**Gender**
Male	238	36.3	226 (95)	12 (5)
Female	418	63.7	392 (93.8)	26 (6.2)
**Age group**
60–62	126	19.2	116 (92.1)	10 (7.9)
63–65	197	30	189 (95.9)	8 (4.1)
66–68	183	27.9	170 (92.9)	13 (7.1)
69 or more	150	22.9	143 (95.3)	7 (4.7)
**Marital status**
Married	583	88.9	552 (94.7)	31 (5.3)
Single	73	11.1	66 (90.4)	7 (9.6)
**Hukou**				
Rural	574	87.5	542 (94.4)	32 (5.6)
Urban	82	12.5	76 (92.7)	6 (7.3)
**Migrated range**
Trans-counties	146	22.3	138 (94.5)	8 (5.5)
Trans-cities	441	67.2	417 (94.6)	24 (5.4)
Trans-provinces	66	10.1	60 (90.9)	6 (9.1)
Trans-countries	3	0.4	3 (100)	0 (0)
**Education level**
Never be educated	196	29.9	183 (29.6)	13 (34.2)
Primary school	144	22	136 (22)	8 (21.1)
Junior high school	192	29.2	181 (29.3)	11 (28.9)
High school or above	124	18.9	118 (19.1)	6 (15.8)
**Migration scale**
Alone	233	35.5	216 (92.7)	17 (7.3)
With one family member	394	60.5	377 (61)	17 (44.7)
With two family members or more	26	4	22 (84.6)	4 (15.4)
**Income**
0–100 yuan per month	221	33.7	209 (94.6)	12 (5.4)
101–500 yuan per month	128	20.7	128 (94.1)	8 (5.9)
501–1,000 yuan per month	111	16.9	106 (95.5)	5 (4.5)
1,001–2,500 yuan per month	93	14.2	86 (92.5)	7 (7.5)
2,501–10,000 yuan per month	95	14.5	89 (93.7)	6 (6.3)

### Association Between Intergenerational Support and Subjective Well-Being

Table 2 shows the social integration characteristics of the 656 participants. Most participants reported that their child had migrated to Jinan over 6 years ago (83.2%), was male (79.4%), often worked overtime from home (68.3%), often worked overtime at his/her company (54.7%), had college education level or above (75%), had terrible conjugal relations (75.2%), and was in good health (97.4%, Table 2).

**Table 2:** Intergenerational support and Children's Household Characteristics among migrants elderly following children.

**Variables**	***N***	**%**	**Migrants' SWB**		**χ^2^**	***P***
			**Yes**	**No**		
Children's duration in the inflow area					1.457	0.483
≤ 3 years	28	4.3	26 (92.9)	2 (7.1)		
3-6 years	82	12.5	75 (91.5)	7 (8.5)		
>6 years	546	83.2	517 (94.7)	29 (5.3)		
Children's gender					8.811	**0.006**
Male	521	79.4	498 (95.6)	23 (4.4)		
Female	135	20.6	120 (96.3)	15 (3.7)		
Work overtime from home					4.72	**0.031**
Often	448	68.3	416 (92.9)	32 (7.1)		
No or less	208	31.7	202 (97.1)	6 (2.9)		
Work overtime at company					1.624	0.240
Often	359	54.7	342 (95.3)	17 (4.7)		
No or less	297	45.3	276 (92.9)	21 (7.1)		
Education level					1.983	0.576
Primary school	14	2.1	13 (92.9)	1 (7.1)		
Junior school	56	8.5	51 (91.1)	5 (8.9)		
Senior school	94	14.3	87 (92.6)	7 (7.4)		
College or above	492	75	467 (94.9)	25 (5.1)		
Conjugal relation of children					13.666	**0.001**
Good	163	24.8	144 (88.3)	19 (11.7)		
Terrible	493	75.2	474 (96.1)	19 (3.9)		
Health condition					0.000	1
Good	639	97.4	602 (94.2)	37 (5.8)		
Terrible	17	2.6	16 (94.1)	1 (5.9)		

*N, Number of samples; %, The percentage of a feature in the variable; Migrants' SWB, MEFC choosing Yes or No in dependent variable measurements; Yes, good SWB; No, poor SWB; χ^2^, represents the resulting value of a chi-square analysis; P, represents the statistical probability*.

Table 2 shows the associations between intergenerational support and SWB among the MEFCs. There were statistically significant differences regarding child's gender (χ^2^ = 8.811, *P* = 0.006), whether the child worked overtime from home (χ^2^ = 4.72, *P* = 0.031), and the quality of the child's conjugal relationship (χ^2^ = 13.666, *P* = 0.001).

### Migrants' Subjective Well-Being and Social Integration

Table 3 shows the social integration characteristics of the 656 participants. Most liked the city in which they were currently living (93.6%), were willing to stay in Jinan for a long time (51.2%), did not have a domiciliary transfer (74.5%), were willing to integrate with the locals (93.7%), had no community participation at all (52.3%), had a basic understanding of the local dialect (50.8%), understood local wedding customs (42.4%), did not understand local funeral customs (41.6%) and understood the local eating customs (51.1%, Table 3).

**Table 3:** Social integration of migrant elderly following children.

**Variables**	***N***	**%**	**Migrants' SWB**		**χ^2^**	***P***
			**Yes**	**NO**		
Favor of current city					20.103	**0.000**
Yes	614	93.6	585 (95.3)	29 (4.7)		
No	42	6.4	33 (78.6)	9 (21.4)		
Willingness to stay for a long time					1.607	0.448
Yes	336	51.2	319 (94.9)	17 (5.1)		
No	215	32.8	199 (92.6)	16 (7.4)		
Not sure	105	16	100 (95.2)	5 (4.8)		
Domiciliary transfer					1.767	0.413
Yes	58	8.8	53 (91.4)	5 (8.6)		
No	489	74.5	460 (94.1)	29 (5.9)		
Not sure	109	16.6	105 (96.3)	4 (3.7)		
Willingness to integrate with the local people					10.198	0.07
Yes	615	93.7	584 (95)	31 (5)		
No	41	6.3	34 (82.9)	7 (17.1)		
Community participation					1.920	0.383
Often	84	12.8	79 (94)	5 (6)		
Sometime	229	34.9	212 (92.6)	27 (7.4)		
Not at all	343	52.3	327 (95.3)	16 (4.7)		
Local dialect					6.114	**0.047**
Fully understand	333	50.8	310 (93.1)	23 (6.9)		
Basic understand	293	44.7	282 (96.2)	11 (3.8)		
Do not understand	30	4.6	26 (86.7)	4 (13.3)		
Wedding custom					3.00	0.223
Fully understand	278	42.4	267 (96)	11 (4)		
Basic understand	102	15.5	95 (93.1)	7 (6.9)		
Do not understand	276	42.1	256 (92.8)	20 (7.2)		
Funeral custom					6.832	**0.033**
Fully understand	263	40.1	255 (97)	8 (3)		
Basic understand	120	18.3	109 (90.8)	11 (9.2)		
Do not understand	273	41.6	254 (93)	19 (7)		
Eating custom					6.288	**0.043**
Fully understand	335	51.1	323 (96.4)	12 (3.6)		
Basic understand	138	21	126 (91.3)	12 (8.7)		
Do not understand	183	27.9	169 (92.3)	14 (7.7)		

*N, Number of samples; %, The percentage of a feature in the variable; Migrants' SWB, MEFC choosing Yes or No in dependent variable measurements; Yes, good SWB; No, poor SWB; χ^2^, represents the resulting value of a chi-square analysis; P, represents the statistical probability*.

We investigated the association between social integration and SWB among MEFCs (Table 3). There were statistically significant differences regarding preference for the current city (χ^2^ = 20.103, *P* = 0.000), understanding of the local dialect (χ^2^ = 6.114, *P* = 0.047), understanding of local funeral customs (χ^2^ = 6.832, *P* = 0.033), and understanding of eating customs (χ^2^ = 6.288, *P* = 0.043).

### Migrants' Subjective Well-Being and Utilization of Medical and Health Services

Table 4 shows the participants' utilization of medical and health services. Most were not ill last year (51.8%), did not have local medical insurance (82.5%), had not experienced medical return (74.5%), had received treatment in the local hospital (76.2%), had used in-patient service in the past year (84.5%), had chosen not to go to hospital in order to avoid troubling their children (95.1%), had not sought treatment for a disease in order to avoid troubling their children (95.7%), had experience purchasing medicine for themselves (88.4%), and had experience of going to the hospital alone (92.1%, Table 4).

**Table 4:** Utilization of medical and health services of migrant elderly following their children.

**Variables**	***N***	**%**	**Migrants' SWB**		**χ^2^**	***P***
			**Yes**	**No**		
Have illness last year					0.321	0.618
Yes	316	48.2	296 (93.7)	20 (6.3)		
No	340	51.8	322 (94.7)	18 (5.3)		
Local medical insurance					0.346	0.515
Yes	115	17.5	107 (93)	8 (7)		
No	541	82.5	511 (94.5)	30 (5.5)		
Medical return					0.329	0.676
Yes	127	19.4	121 (95.3)	6 (4.7)		
No	529	80.6	497 (94)	32 (6)		
Diseased treatment					0.71	0.701
Homeward treatment	127	19.4	121 (95.3)	6 (4.7)		
Treatment in local hospital	500	76.2	469 (93.8)	31 (6.2)		
Without any treatment	19	4.4	28 (96.6)	1 (3.4)		
Had in-patient service in the past year					13.928	**0.001**
Yes	554	84.5	530 (95.7)	24 (4.3)		
No	102	15.5	88 (86.3)	14 (13.7)		
Do not go to the hospital for fear of troubling children					15.944	**0.001**
Yes	624	95.1	593 (95)	31 (5)		
No	32	4.9	25 (78.1)	7 (21.9)		
Do not treat the disease for fear of troubling children					13.103	**0.004**
Yes	628	95.7	596 (94.9)	32 (5.1)		
NO	28	4.3	22 (78.6)	6 (21.4)		
Self-purchase medicine					5.764	**0.023**
Yes	580	88.4	551 (95)	29 (5)		
NO	76	11.6	67 (71.6)	9 (4.4)		
Go to hospital alone					13.723	**0.002**
Yes	604	92.1	575 (95.2)	29 (4.8)		
No	52	7.9	43 (82.7)	9 (17.3)		

*N, Number of samples; %, The percentage of a feature in the variable; Migrants' SWB, MEFC choosing Yes or No in dependent variable measurements; Yes, good SWB; No, poor SWB; χ^2^, represents the resulting value of a chi-square analysis; P, represents the statistical probability*.

As shown in Table 4, factors that had a significant impact on SWB included had in-patient service in the past year (χ^2^ = 13.928, *P* = 0.001), choosing not to go to hospital in order to avoid troubling one's children (χ^2^ = 15.944, *P* = 0.001), not seeking treatment for a disease in order to avoid troubling one's children (χ^2^ = 13.103, *P* = 0.004), purchasing medicine for oneself (χ^2^ = 5.764, *p* = 0.023), and going to the hospital alone (χ^2^ = 13.723, *P* = 0.002).

### Factors Associated With Migrants' Subjective Well-Being

In this section, we present our results from the three models created to better explore the association between intergenerational support, social integration, and SWB (Table 5). Coefficients in Model 1 showed that child's gender, child working overtime from home, and quality of child's conjugal relationship were significantly related to migrants' SWB. However, compared to migrants who followed male children, migrants whose children were female had relatively poor SWB (*P* = 0.011, OR = 0.408, 95%CI: 0.204, 0.818). Moreover, having a child with a poor conjugal relationship was significantly negatively related to migrants' SWB (*P* = 0.001, OR = 0.322, 95%CI: 0.164, 0.630).

**Table 5:** Factors associated with migrants' SWB.

**Variables**	**Model 1** **OR (95%CI)**	**Model 2** **OR (95%CI)**	**Model 3** **OR (95%CI)**
**Intergenerational support (Characteristics of children)**
Children's gender of older people			
Male	1	1	1
Female	0.408 (0.204,0.818)^*^	0.354 (0.166,0.756)^*^	0.401 (0.180,0.893)^*^
**Work overtime from home**
Often	1	1	1
No or less	6.511 (0.877,48.338)	8.132 (1.017,65.032)^*^	7.972 (0.991,64.142)
**Conjugal relation**
Good	1	1	1
Terrible	0.322 (0.164,0.630)^***^	0.246 (0.115,0.524) ^***^	0.223 (0.099,0.504)^***^
**Social integration**
**Favor of current city**
No		1	1
Yes		5.484 (1.878,16.015)^**^	5.358 (1.631,17.599)^**^
**Willingness to integrate with the local people**
No		1	1
Yes		2.903 (0.853,9.883)	3.648 (0.986,13.514)
**Local dialect**
Fully understand		1	1
Basic understand	1	3.064 (1.338,7.016)^**^	2.869 (1.203,6.843)^**^
Do not understand		1.424 (0.356,5.698)	1.110 (0.258,4.783)
**Funeral custom**
Fully understand		1	1
Basic understand		0.256 (0.067,0.981)^*^	0.259 (0.060,1.116)
Do not understand		0.637 (0.177,2.296)	0.921 (0.231,3.669)
**Eating custom**
Fully understand		1	1
Basic understand		1.045 (0.313,3.489)	1.033 (0.267,3.998)
Do not understand		0.734 (0.216,2.494)	0.656 (0.174,2.476)
**Utilization of medical and health services**
**Had used in-patient service**
No			1
Yes			0.216 (0.094,0.497)^***^
**Do not go to the hospital for fear of troubling children**
No			1
Yes			0.195 (0.034,1.113)
**Do not treat the disease for fear of troubling children**
Yes			1
NO			1.088 (0.159,7.450)
**Self-purchase medicine**
Yes			1
NO			2.360 (0.471,11.835)
**Go to hospital alone**
Yes			1
No			0.242 (0.043,1.346)

* *P <0.05*,

** *P <0.01*,

*** *P <0.001*.

In Model 2, we included social integration status. The results showed that, among MEFCs, the association between social integration and SWB was statistically significant. Specifically, the migrants who liked their current city (*P* =0.002, OR = 5.484, 95%CI: 1.878, 16.015) who had a basic understanding of the local dialect (*P* =0.008, OR = 3.064, 95%CI: 1.338,7.016) were more likely to have good SWB. And MEFCs who had a basic understanding of the funeral custom (*P* =0.047, OR = 0.256, 95%CI: 0.067, 0.981) were likely to have poor SWB.

Model 3 showed that intergenerational support, social integration, and had used in-patient service were statistically associated with SWB. Specifically, MEFCs whose child was female (*P* = 0.025, OR = 0.401, 95%CI: 0.180,0.893), whose child had a terrible conjugal relationship (*P* <0.001, OR = 0.223, 95%CI: 0.099, 0.504), and who had used in-patient service in the past year (*P* <0.01, OR = 0.216 95%CI: 0.094, 0.497) were more likely to have poor SWB, but those who liked their current city (*P* = 0.006, OR = 5.358, 95%CI: 1.631,17.599) and those who had a basic understanding of the local dialect (*P* =0.017, OR = 2.869, 95%CI: 1.203, 6.843) were more likely to have good SWB.

## Discussion

### Subjective Well-Being of Migrant Elderly Following Children in Jinan, China

Our study showed that ~94.2% of MEFCs have high SWB. The rate of good SWB among our sample of MEFCs was higher than that reported in the 2014 China Family Panel Study (71.4%) (60). A possible reason for this difference was that all 656 participants had grandchildren. It is also possible that taking care of grandkids reduces the feeling of loneliness and gives the adults a distraction. And hence they are less likely to feel depressed and lonely which would translate to a poor SWB (14, 61, 62).

### Association Between the Independent Variables and the Subjective Well-Being of Migrant Elderly Following Children

#### Association Between Intergenerational Support and Subjective Well-Being

This study identified several factors that have a statistically significant influence on MEFCs' SWB. These included the gender of one's child, whether the child works overtime from home, and the quality of the child's conjugal relationship. These findings echo those of previous studies that also suggested that the gender of one's child (26, 51), whether one's child works overtime from home (50), and the quality of a child's conjugal relationship (26, 51) influence migrants' SWB.

The SWB of migrant older adults who had followed their daughters was found to be lower than that of migrant older adults who had followed their sons. This is similar to other studies' findings. A previous study reported that male offspring are considered more valuable to parents than female offspring (63), while another China-based study found that sons are associated with better SWB among mothers (64). A possible reason for these findings is that, in traditional societies with incomplete financial markets there is a lack of family retirement function products, meaning many important resources, such as medical care and monetary support, are accessible only through the assistance of social networks, such as families. Most of our research subjects had migrated from rural areas to cities, and most did not have a regular income. In this context, male offspring would take on more of the family old-age obligations, thus, providing better intergenerational support (65, 66). For example, in traditional agricultural practices, men have a higher production capacity than women (67). Further, in Shandong, where Confucianism has a wide influence, the concept of patriarchalism is deeply rooted. Notably, Lu reported that, at times when a child is marrying or preparing for the birth of his/her own child, sons are associated with significantly lower SWB among parents than daughters, and parents also lose happiness when “helping children buy a house” which cause the huge economic burden (47). However, removing the impact of economic burdens reveals that the SWB of migrant parents of boys is higher than that of migrant parents of girls.

Second, this study found that MEFCs whose children did not work overtime or who worked overtime infrequently had good SWB (Model 1). These results are similar to those reported by He (28), who found that having children who frequently work overtime from home decreases the SWB of MEFCs. A possible reason for this is that children who regularly work overtime have less time to provide daily care for their parents, and this lack of daily care negatively affects the parents' quality of life in their later years. Another possible explanation for this is that MEFCs may view their primary role as helping their children care for their families. However, if MEFCs are required to adopt the role of parents to their grandchildren, intra-role conflicts can easily ensue. Such dual adoption of roles is not only difficult for MEFCs, but can also become a burden in life and may hinder family integration (68).

Third, the present results showed that MEFCs whose children had good conjugal relationships had better SWB than other MEFCs. This also agrees with earlier studies' observations. Prior studies have noted that children's conjugal relationships have a positive impact on the SWB of older adults, and also underlined the importance of a good family atmosphere (69). Meanwhile, Liu highlighted that infrequent family reunifications and the occurrence of family conflicts lead to a decline in older adults' SWB (14). A possible reason for this finding is that the conjugal relationships of children greatly affect children's mood and the family atmosphere. These two factors directly affect the emotional support older adults receive. Notably, Xu mentioned that children's attitudes toward their parents affect their parents' SWB (70). When there are conflicts between children and their spouses, the parents must adjust their lifestyles to accommodate such tensions. Migrants may need to completely change their lifestyles (when compared to their pre-migration habits), thereby reducing their SWB.

#### Association Between Social Integration and Subjective Well-Being

This study examined the impact of social integration on the SWB of MEFCs through considering the dimensions of identification and acculturation. First, MEFCs who liked their current city had better SWB. This finding supported that of another China-based study (52). A possible explanation for this is that different cities have different characteristics or personalities, meaning each city has a unique influence on new residents (71). The urban integration of migrants is an interactive process between migrants and the local society (56), and the uniqueness of a city can have both positive and negative effects on migrants. Positive effects could result in migrants integrating strongly into the city and being accepted by the locals, creating a sense of solidarity and pride (39). Meanwhile, the negative effects could result in migrants finding the city repulsive. The relationship between urban features and migrants' characteristics relied on their selection mechanisms. Cities alienated populations with a low level of urban awareness, and this is not conducive to population integration (39, 72, 73). Different cities attracted different migrants. For example, when children migrated to a particular city, their families, through contact with the child, develop a certain understanding of the local area, which can accelerate their subsequent integration. One of the possible reasons was that young people who chose to migrate to cities such as Beijing and Shanghai usually suffered from heavier work pressure and had longer working time. It might reduce the time of daily care for their parents, which influenced the SWB level of MEFC. However, those young people in Shandong Province who chose to migrate to Jinan may have less life stress compared to those people in first-tier cities. Therefore, they could have more time to take care of their parents. Another possible reason could be explained from the MEFC's perspective. Those MEFC who lived in first-tier cities would face more problems in getting used to the lifestyle of modern cities, which might related to poor social integration (50).

Second, this study found that the older people who had a basic understanding of the local dialect had a higher SWB level than those who had a full understanding and those who did not understand the local dialect at all. This is also consistent with previous research findings. A study has indicated that understanding dialects impacts social integration with cities (74). Further, Leng mentioned that SWB decreases as one's familiarity with a dialect grows, with greater familiarity with dialects indicating longer time spent as a migrant (75). A possible explanation for our finding is that, compared to other migrants, older migrants arrive in a new city for a relatively short time and may have a greater interest in the local culture of Jinan. Having a basic understanding of the local dialect helps migrants establish new social relationships, and these social relationships might facilitate the acquiring of different kinds of social support that affect the MEFCs' SWB (60). However, strong familiarity with the dialect indicates a long migration duration. Such a long duration may be associated with more family and social conflicts, which consequently leads to a decrease in MEFCs' SWB.

#### Association Between Used In-patient Service in the Past Year and Subjective Well-Being

This study found that MEFCs who had used in-patient service in the past year were more likely to have poor SWB than those who had not used in-patient service in the past year. This is consistent with previous findings (76). Some studies have suggested that long-term in-patient service influences migrants' SWB (77, 78). Although the physical symptoms of long-term hospitalized patients gradually disappear through drug treatments and psychological interventions, they usually create a “stigma” (5), which can affect the patients' psychological, physical, and social life, leading to poor SWB (6).

### Strengths

First, few studies have focused on MEFCs. Compared with native older people, MEFCs represent a vulnerable group because of less social integration and family support. Further, the self-report method used in the present study for measuring the MEFCs' SWB is known to be a reliable and valid means of measuring SWB (46).

### Limitations

This study contained several limitations. First, the data were cross-sectional. Therefore, causal relationships could not be determined. After follow-up data collection in the future, we will analyze the longitudinal data and determine the causality of variables. Second, the questionnaire lacked systematic scales for measuring SWB, social integration, and intergenerational support. Thus, we included in the questionnaire only a small number of items relevant to the target variables. Further research should seek to bridge this gap. Third, the data on the utilization of medical and health services were sourced from self-report items, meaning recall bias may be present. Fourth, the survey did not measure the intergenerational support received by children. Intergenerational support is a two-way construct (69). The present researchers plan to conduct further research (in the MEFC context) on this two-way characteristic of intergenerational support. Finally, our study subjects were MEFCs who lived in Jinan, so it is not known whether our results are applicable to MEFCs who lived in other regions.

## Conclusion

To our knowledge, this was the first study to examine the determinants of SWB among Chinese MEFCs from the perspective of intergenerational support and social integration. We found that intergenerational support was associated with SWB among MEFCs. MEFCs who have a son, whose children have a good conjugal relation were more likely to have good SWB. Further, social integration was related to SWB among MEFCs, which included the two dimensions: identification and acculturation. Meanwhile, MEFCs with good social integration were more likely to have good SWB. Based on these findings, the policy-maker may consider MEFCs as a vulnerable group among migrants, targeting promotion in intergenerational support and social integration among MEFCs. The government could make it possible for children of MEFCs to spend more time with them through measures such as limiting overtime hours. In this way, MEFCs could receive more intergenerational support from their children. Meanwhile, the government should consider creating senior activity centers to create more social opportunities for seniors and accelerate their adaptation to the current city throughnetworking, with the aim to improving the social integration of MEFCs.

## Data Availability Statement

The original contributions presented in the study are included in the article/supplementary material, further inquiries can be directed to the corresponding author/s.

## Ethics Statement

The studies involving human participants were reviewed and approved by the Ethical Committee of Shandong University (No. 20180225). The patients/participants provided their written informed consent to participate in this study.

## Author Contributions

QJ analyzed the data and drafted the manuscript. FK applied the fund to support this study, designed the study, completed the questionnaire design, supervised and joined the data collection, instructed the writing, statistical analysis, data processing, and gave comments on the modification of manuscript. SL gave many valuable comments on the draft and also polished it. All authors read and approved the final manuscript.

## Funding

This study was supported and funded by National Natural Science Foundation of China (No. 71804094), China Postdoctoral Science Foundation (No. 2016M592161), Natural Science Foundation of Shandong Province (No. ZR2016GB02), Postdoctoral Science Foundation of Shandong Province (No. 201603021), and the Fundamental Research Funds of Shandong University (Nos. 2015HW002 and 2018JC055). The research team greatly appreciate the funding support, and the research participants for their corporation and support.

## Conflict of Interest

The authors declare that the research was conducted in the absence of any commercial or financial relationships that could be construed as a potential conflict of interest.

## Publisher's Note

All claims expressed in this article are solely those of the authors and do not necessarily represent those of their affiliated organizations, or those of the publisher, the editors and the reviewers. Any product that may be evaluated in this article, or claim that may be made by its manufacturer, is not guaranteed or endorsed by the publisher.
